# Effect of ventilator-associated tracheobronchitis on outcome in patients without chronic respiratory failure: a case–control study

**DOI:** 10.1186/cc3508

**Published:** 2005-03-31

**Authors:** Saad Nseir, Christophe Di Pompeo, Stéphane Soubrier, Hélène Lenci, Pierre Delour, Thierry Onimus, Fabienne Saulnier, Daniel Mathieu, Alain Durocher

**Affiliations:** 1 Intensive Care Unit, Calmette Hospital, Regional University Centre, and Medical Assessment Laboratory, EA 3614, Lille II University, Lille, France; 2 Medical Assessment Laboratory, EA 3614, Lille II University, Lille, France; 3 Intensive Care Unit, Calmette Hospital, Regional University Centre, Lille, France

## Abstract

**Introduction:**

Our objective was to determine the effect of ventilator-associated tracheobronchitis (VAT) on outcome in patients without chronic respiratory failure.

**Methods:**

This was a retrospective observational matched study, conducted in a 30-bed intensive care unit (ICU). All immunocompetent, nontrauma, ventilated patients without chronic respiratory failure admitted over a 6.5-year period were included. Data were collected prospectively. Patients with nosocomial pneumonia, either before or after VAT, were excluded. Only first episodes of VAT occurring more than 48 hours after initiation of mechanical ventilation were studied. Six criteria were used to match cases with controls, including duration of mechanical ventilation before VAT. Cases were compared with controls using McNemar's test and Wilcoxon signed-rank test for qualitative and quantitative variables, respectively. Variables associated with a duration of mechanical ventilation longer than median were identified using univariate and multivariate analyses.

**Results:**

Using the six criteria, it was possible to match 55 (87%) of the VAT patients (cases) with non-VAT patients (controls). *Pseudomonas aeruginosa *was the most frequently isolated bacteria (34%). Although mortality rates were similar between cases and controls (29% versus 36%; *P *= 0.29), the median duration of mechanical ventilation (17 days [range 3–95 days] versus 8 [3–61 days]; *P *< 0.001) and ICU stay (24 days [range 5–95 days] versus 12 [4–74] days; *P *< 0.001) were longer in cases than in controls. Renal failure (odds ratio [OR] = 4.9, 95% confidence interval [CI] = 1.6–14.6; *P *= 0.004), tracheostomy (OR = 4, 95% CI = 1.1–14.5; *P *= 0.032), and VAT (OR = 3.5, 95% CI = 1.5–8.3; *P *= 0.004) were independently associated with duration of mechanical ventilation longer than median.

**Conclusion:**

VAT is associated with longer durations of mechanical ventilation and ICU stay in patients not suffering from chronic respiratory failure.

## Introduction

Nosocomial lower respiratory tract infections are the most common nosocomial infections in the intensive care unit (ICU) [1]. Although several studies have investigated nosocomial pneumonia, few evaluated ventilator-associated tracheobronchitis (VAT).

VAT is a common nosocomial infection among mechanically ventilated patients. VAT rates of 3.7–10.6% have been reported in the literature [2-4]. In a previous descriptive prospective cohort study conducted in 2128 patients [4], our group demonstrated that VAT was associated with increased durations of mechanical ventilation and ICU stay. However, two major limitations of the study prevented us from drawing definite conclusions: absence of adjustment for duration of mechanical ventilation before the occurrence of VAT; and inclusion of patients with and patients without chronic respiratory failure. Therefore, we performed a retrospective case–control study to assess the effect of VAT on outcomes in patients without chronic respiratory failure.

## Methods

This retrospective case–control study was conducted in our 30-bed ICU from March 1993 to September 1999. Because it was observational, institutional review board approval was not required, which is in accordance with institutional review board regulations.

All immunocompetent, nontrauma patients without chronic respiratory failure who were intubated and ventilated for more than 48 hours were eligible. Patients with chronic respiratory failure, trauma patients, patients who were not ventilated or ventilated for less than 48 hours, patients who received only noninvasive pressure ventilation, patients with tracheostomy at ICU admission and immunocompromised patients were not eligible. Patients who developed nosocomial pneumonia, before or after the occurrence of VAT, were excluded. The patients included in the present study were also included in our previous prospective observational study of VAT [4], representing 5% of the 2128 patients included in the previous study.

Patients were intubated via either the oral or the nasal route, according to clinical status and preference of the physician in charge. The oropharyngeal cavity was cleaned four times daily with chlorhexidine solution. Continuous subglottic suctioning was not utilized. The ventilator circuit was not changed routinely. In all patients a heat–moisture exchanger was positioned between the Y-piece and the patient; the heat–moisture exchangers were changed every 48 hours, or more frequently if they were visibly soiled. No patient received inhaled antibiotics. Patients were kept in a semirecumbent position during most of their period of mechanical ventilation. Sedation and weaning procedures were done at the discretion of the physician in charge. No systematic stress ulcer prophylaxis and no selective digestive decontamination was given. Tracheal aspiration was performed by nurses every 3 hours and whenever necessary.

Throughout the study, endotracheal aspirates for quantitative bacterial cultures were obtained routinely on admission, weekly thereafter, and whenever VAT or ventilator-associated pneumonia (VAP) was suspected. Antimicrobial therapy for VAT was at discretion of the physician in charge.

All data were collected prospectively. VAT episodes were identified by prospective surveillance of nosocomial infections. Only first episodes of VAT occurring more than 48 hours after initiation of mechanical ventilation were included. 'Cases' are VAT patients, and 'controls' are patients without VAT. Tracheobronchitis was defined using all of the following criteria: fever (>38°C) with no other recognizable cause; new or increased sputum production; positive (≥ 10^6 ^colony-forming units/ml) endotracheal aspirate culture [5], yielding a new bacteria; and no radiographic evidence of nosocomial pneumonia. In patients with abnormal chest radiograph at admission, the absence of new or progressive radiographic infiltrates was required. To define nosocomial pneumonia, a second set of criteria developed by the US Centers for Disease Control and Prevention was used [6]. Other nosocomial infections were defined using the Centers for Disease Control and Prevention criteria [6].

Antimicrobial therapy was deemed adequate when at least one antibiotic active *in vitro *on all organisms causing VAT was administrated at an appropriate dosage within the first 48 hours after VAT was identified. Chronic respiratory failure was defined by the presence of chronic obstructive pulmonary disease [7] or chronic restrictive pulmonary disease diagnosed on the basis of history, physical examination, chest radiography and respiratory function tests. Immunosupression was defined as the presence of neutropenia (leucocyte count <1000/μL or neutrophils <500/μL), long-term corticosteroid therapy (≥ 0.5 mg/kg per day for more than 1 month), or HIV infection (CD4^+ ^cell count <50/μL for the previous 6 months). Multidrug-resistant bacteria were defined as methicillin-resistant *Staphylococcus aureus*, ceftazidime or imipenem-resistant *Pseudomonas aeruginosa, Acinetobacter baumannii*, extended-spectrum β-lactamase-producing Gram-negative bacilli, and *Stenotrophomonas maltophilia*. Prior antibiotic treatment was defined as any antibiotic treatment over the 2 weeks preceding ICU admission. Outcomes evaluated included ICU mortality, and durations of mechanical ventilation and ICU stay.

Each case patient was matched to one control patients according to all the following criteria: duration of mechanical ventilation before VAT occurrence (a control patient had to have been mechanically ventilated for at least as long as a case patient had before they developed VAT); primary diagnosis for admission; category of admission (medical/surgical); Simplified Acute Physiology Score II on admission (± 5 points) [8]; age (± 5 years); and date of admission (when more than one potential control was well matched to a case).

### Statistical analysis

SPSS software (SPSS Institute Inc., Chicago, IL, USA) was used to analyze the data. Cases were compared with controls using McNemar's test for qualitative variables, and Wilcoxon's signed-rank test for quantitative variables.

Because the distribution of duration of mechanical ventilation was skewed, we first determined the median duration of mechanical ventilation in cases and controls, and then we performed univariate and multivariate analyses to identify those variables associated with duration of mechanical ventilation longer than median. The following variables were included in univariate analysis: age, sex, Simplified Acute Physiology Score II on admission, transfer from other wards, diabetes mellitus, primary diagnosis for admission, organ failures [9], antibiotic use, tracheostomy, VAT related to multidrug-resistant bacteria, and VAT. A stepwise logistic regression, including significant (*P *< 0.05) variables, was used to determine which variables were independently associated with duration of mechanical ventilation longer than median.

In order to determine the impact of antibiotic administration on VAT patient outcome, case patients receiving adequate antibiotic treatment were compared with those who received inadequate antibiotic treatment.

Proportions were compared using the χ^2 ^test or the Fisher's exact test where appropriate; continuous variables were compared using the Mann–Whitney U-test.

## Results

**Figure 1:**
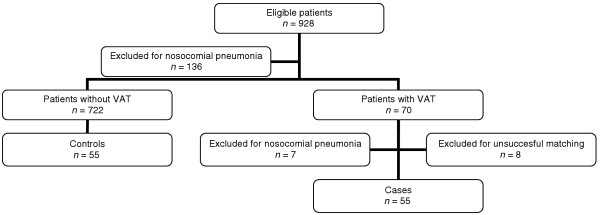
Study profile. VAT, ventilator-associated tracheobronchitis.

A total of 928 patients were eligible, 136 (14%) of whom were excluded because they developed nosocomial pneumonia before VAT. Seventy (8%) first episodes of VAT were diagnosed in the 792 remaining patients. Seven of the 70 patients (10%) were excluded because they subsequently developed nosocomial pneumonia. Using the six criteria outlined above (see Methods), it was possible to match 55 (87%) of the VAT patients without prior or subsequent nosocomial pneumonia (cases) with non-VAT patients (controls; Fig. 1).

Before ICU admission and during the ICU stay, cases received antibiotics more frequently than did controls. During the ICU stay tracheostomy was performed more frequently in cases than in controls. Other patient characteristics were similar between case and control patients (Table 1). The mean period between ICU admission and development of VAT was 11 ± 8 days (median 8 [range 3–47] days). The mean period between starting mechanical ventilation and development of VAT was 10 ± 9 days (median 7 [range 3–47] days).

A total of 86 micro-organisms were isolated in the 55 VAT episodes. The more frequently isolated bacteria were *P aeruginosa *(34%), *A baumannii *(18%) and methicillin-resistant *S aureus *(11%). Thirty (54%) VAT episodes were polymicrobial, and 31 (56%) were related to multidrug-resistant bacteria (Table 2).

Although the durations of mechanical ventilation and ICU stay were significantly longer in cases than in controls, no significant difference was found in mortality rate between case and control patients (Table 3). No significant difference in outcome was found between VAT patients who received adequate antibiotic treatment and those who received inadequate antibiotic treatment (Table 4). In cases with multidrug-resistant bacteria compared with cases with other bacteria, we observed similar durations of mechanical ventilation (23 ± 17 days versus 18 ± 13 days; *P *= 0.869), similar lengths of ICU stay (29 ± 14 versus 29 ± 18 days; *P *= 0.166) and similar ICU mortality rates (10/31 [32%] versus 6/24 [25%]; *P *= 0.359).

The results of univariate and multivariate analyses are presented in Table 5.

## Discussion

The results of this study demonstrate that VAT is associated with increased duration of mechanical ventilation and ICU stay in immunocompetent nontrauma patients without chronic respiratory failure.

Tracheobronchitis is characterized by lower respiratory tract inflammation and increased sputum production. These factors may generate weaning difficulties, resulting in longer duration of mechanical ventilation. Extubation failure and difficult weaning have been reported to be associated with increased sputum volume in mechanically ventilated patients [10].

Previous studies [4,11] highlighted the link between tracheobronchitis and prolonged duration of mechanical ventilation, but these studies did not adjust for confounding factors; in particular, they did not adjust for duration of mechanical ventilation before development of VAT. Thus, based on those studies VAT could be considered a cause or a consequence of prolonged mechanical ventilation. The present case–control study, in which we adjusted for several confounding factors, is to our knowledge the first to demonstrate that VAT is independently associated with longer duration of mechanical ventilation in patients without chronic respiratory failure. However, an interventional randomized study is needed to confirm our findings.

In this study, duration of ICU stay was significantly longer in cases than in controls. However, mortality rates were similar between the two groups. In contrast, a recent prospective observational study [3], conducted in patients who had undergone heart surgery, found significantly higher mortality rates in patients with VAT than in noncolonized patients (20.7% versus 1.6%), and no significant difference in ICU and hospital lengths of stay between the two groups (12 days versus 5 days, and 20 days versus 13 days, respectively). However, the number of patients with VAT included in that study was small (*n *= 29). In addition, VAT patients who developed subsequent VAP were not excluded. Moreover, no adjustment was made for confounding factors.

VAT is probably an intermediate process between lower respiratory tract colonization and VAP. The diagnosis of VAT may be difficult in patients with chest radiographic abnormalities at ICU admission. However, recent guidelines recommend using new chest radiograph infiltrates as a criterion for diagnosis of VAP [12]. On the other hand, VAT is also difficult to differentiate from colonization. However, only new bacteria were taken into account in the present study. Moreover, we used quantitative tracheal aspirates to diagnose VAT, with a high threshold at 10^6 ^colony-forming units/ml.

The high proportion of multidrug-resistant bacteria in patients with VAT may be accounted for by the following factors: 87% of these patients were transferred from other wards; 72% of patients with VAT received antibiotics before ICU admission; and there was a long mean period between ICU admission and VAT development. These factors are well known to be associated with the emergence of multidrug-resistant bacteria in ICU patients [13].

Whether antibiotics should be administered to patients with VAT is actually a subject of debate. Clinical practice with respect to antibiotic treatment in patients with VAT varies widely between ICU physicians. Whereas some physicians do not treat this infection, considering it to be simple colonization, others routinely treat patients with VAT or only those patients with weaning difficulties and/or underlying disease [11,14,15]. In the present study only 21% of patients with VAT received antibiotics to treat this infection. No significant difference in outcome was found between patients who received adequate antimicrobial treatment and those with inadequate antimicrobial treatment. However, our findings are limited by the small number of VAT patients who received adequate antibiotic treatment. Antibiotic treatment could eradicate respiratory bacterial load and decrease sputum production. In a prospective study conducted in long-term mechanically ventilated patients with chronic bacterial colonization, Palmer and coworkers [11] observed a significant decrease in tracheal secretion volume, inflammatory cells and soluble intercellular adhesion molecule-1 burden in those patients who received antibiotics. Nevertheless, excessive antibiotic usage is associated with subsequent emergence of multidrug-resistant bacteria and causes measurable harm in ICU patients [16,17]. Therefore, further randomized studies are warranted to determine whether patients with VAT should be treated with antibiotics [18].

Recent guidelines on appropriate antibiotic use for treatment of acute respiratory tract infections in adults [19] indicate that antibiotic treatment of uncomplicated acute bronchitis should not be routinely applied. This recommendation is based on several randomized controlled studies [20-25] and recent meta-analyses [26-30]; all studies reported no impact of antibiotic treatment on illness duration, activity limitation, or work loss, and all concluded that routine antibiotic treatment of adults with acute bronchitis is not justified. However, all of those studies were conducted in healthy adults. To our knowledge, no randomized controlled study has been reported in mechanically ventilated patients with nosocomial tracheobronchitis.

Our study has several limitations. First, the study was a retrospective analysis of prospectively collected data. Second, our study was performed in a single ICU, and the results may not be applicable to patients in other ICUs. Third, some of the trends observed in the study might have reached statistical significance if the study sample had been larger. Forth, over the long period of study, some changes in case-mix, medical and nursing practices, workload and workforce might have occurred. However, VAT was independently associated with longer than median duration of mechanical ventilation in case and control patients during the study period. Finally, that patients with VAT who subsequently developed VAP were excluded probably overlooked an important consequence of VAT. However, VAP is associated with increased morbidity and mortality, and so exclusion of these patients allowed us to assess the true impact of VAT on outcome [31].

## Conclusion

VAT is associated with increased duration of mechanical ventilation and ICU stay in immunocompetent nontrauma patients without chronic respiratory failure. Further studies are required to confirm our results and to evaluate the impact of antibiotic treatment on outcomes of patients with VAT.

## Key messages

• 	VAT is associated with increased duration of mechanical ventilation and ICU stay in immunocompetent nontrauma patients without chronic respiratory failure.

• 	There was no significant difference in outcome between VAT patients who received adequate antibiotic treatment and those who received inadequate antibiotic treatment.

• 	Further studies are needed to evaluate the impact of antibiotic treatment on outcomes in patients with VAT.

## Abbreviations

ICU = intensive care unit; VAP = ventilator-associated pneumonia; VAT = ventilator-associated tracheobronchitis.

## Competing interests

The author(s) declare that they have no competing interests.

## Figures and Tables

**Table 1:** Patient characteristics

Parameter/characteristic	Cases (*n *= 55)	Controls (*n *= 55)
At admission		
Age (years; mean ± SD]	59.9 ± 18.2	60.3 ± 17.5
Male (*n *[%])	31 (56)	33 (60)
SAPS II (mean ± SD)	37.2 ± 15.3	37.4 ± 14.7
Transfer from other wards (*n *[%])	48 (87)	49 (89)
Diabetes mellitus (*n *[%])	12 (21)	13 (23)
Prior antibiotic treatment* (*n *[%])	40 (72)	15 (27)
Admission category (*n *[%])		
Medical	39 (70)	39 (70)
Surgical	16 (29)	16 (29)
Primary diagnosis for admission (*n *[%])		
Community-acquired pneumonia	12 (21)	12 (21)
Cellulitis	12 (21)	12 (21)
Septic shock	10 (18)	10 (18)
Congestive heart failure	7 (12)	7 (12)
Peritonitis	4 (7)	4 (7)
Acute respiratory distress syndrome	4 (7)	4 (7)
Other	6 (10)	6 (10)
Organ failure (*n *[%])		
Cardiac	11 (20)	17 (30)
Respiratory	38 (69)	41 (74)
Renal	14 (25)	10 (18)
Neurologic	12 (21)	19 (34)
Digestive	3 (5)	5 (9)
During hospitalization		
Tracheostomy^† ^(*n *[%])	12 (21)	5 (9)
Antibiotic treatment^‡^		
*n *(%)	55 (100)	43 (78)
Duration (days; mean ± SD)	13.3 ± 11.5	5.8 ± 9.2
Antibiotic days/1000 ICU-days	485	330

*P = 0.006, ^†^*P *= 0.056 and ^‡^*P *< 0.001 (cases/controls) by univariate analysis. ICU, intensive care unit; SAPS, simplified acute physiology score; SD, standard deviation.

**Table 2:** Bacteria associated with 55 episodes of ventilator-associated tracheobronchitis

Micro-organism	*n *(%)
Gram-negative	66 (76)
*Pseudomonas aeruginosa*	30 (34)
*Acinetobacter baumannii*	16 (18)
*Serratia *spp.	6 (6)
*Enterobacter *spp.	4 (4)
*Escherichia coli*	4 (4)
*Stenotrophomonas maltophilia*	3 (3)
*Klebsiella *spp.	3 (3)
Gram-positive	20 (23)
MRSA	10 (11)
MSSA	5 (5)
*Streptococcus pneumoniae*	5 (5)

MRSA, methicillin-resistant *Staphylococcus aureus*; MSSA, methicillin-sensitive *Staphylococcus aureus*.

**Table 3:** Outcomes of patients with (cases) and patients without (controls) ventilator-associated tracheobronchitis

Outcome	Cases (*n *= 55)	Controls (*n *= 55)	*P*^a^
Duration of mechanical ventilation (days)			
Median (range)	17.0 (3–95)	8.0 (3–61)	<0.001
Mean ± SD	21.6 ± 16.0	13.3 ± 13.1	
Length of ICU stay (days)			
Median (range)	24.5 (5–95)	12.0 (4–74)	<0.001
Mean ± SD	28.0 ± 15.7	17.6 ± 16.6	
ICU mortality (*n *[%])	16 (29)	20 (36)	0.294

^a^Results by univariate analysis. ICU, intensive care unit; SD, standard deviation.

**Table 4:** Impact of antibiotic treatment on outcomes of patients with ventilator-associated tracheobronchitis

Outcome	Antibiotic treatment		*P*^a^
		
	Adequate (*n *= 12)	Inadequate (*n *= 43)	
Duration of mechanical ventilation (days)			
Median (range)	17.0 (3–95)	18.5 (3–58)	0.833
Mean ± SD	18.8 ± 9.7	22.3 ± 17.2	
Length of ICU stay (days)			
Median (range)	22.0 (5–95)	25.0 (6–62)	0.344
Mean ± SD	24.8 ± 14.5	30.5 ± 16.8	
ICU mortality (*n *[%])	5 (41)	11 (25)	0.178

Inadequate antibiotic treatment was given for infectious diseases other than ventilator-associated tracheobronchitis. ^a^Results by univariate analysis. ICU, intensive care unit; SD, standard deviation.

**Table 5:** Factors associated with duration of mechanical ventilation longer than median (14 days) in patients with (cases) and without (controls) ventilator-associated tracheobronchitis

Factor	Univariate analysis			Multivariate analysis	
	
	Number of patients (*n *= 110)	Number of patients with MV duration ≥ 14 days (%)	*P*	OR (95% CI)	*P*
Renal failure on ICU admission					
Yes	24	18 (75)	0.002	4.9 (1.6–14.6)	0.004
No	86	34 (39)			
Tracheostomy					
Yes	17	13 (76)	0.009	4.0 (1.1–14.5)	0.032
No	93	39 (41)			
VAT related to multidrug-resistant bacteria					
Yes	31	22 (70)	0.002	-	-
No	79	30 (37)			
VAT					
Yes	55	35 (63)	0.001	3.5 (1.5–8.3)	0.004
No	55	17 (30)			

CI, confidence interval; ICU, intensive care unit; MV, mechanical ventilation; OR, odds ratio; VAT, ventilator-associated tracheobronchitis.
